# Beyond Sector Retinitis Pigmentosa: Expanding the Phenotype and Natural History of the Rhodopsin Gene Codon 106 Mutation (Gly-to-Arg) in Autosomal Dominant Retinitis Pigmentosa

**DOI:** 10.3390/genes12121853

**Published:** 2021-11-23

**Authors:** Brian G. Ballios, Emily M. Place, Luis Martinez-Velazquez, Eric A. Pierce, Jason I. Comander, Rachel M. Huckfeldt

**Affiliations:** 1 Ocular Genomics Institute, Department of Ophthalmology, Massachusetts Eye and Ear, Harvard Medical School, Boston, MA 02114, USA; brian.ballios@mail.utoronto.ca (B.G.B.); emily_place@meei.harvard.edu (E.M.P.); luis_martinezvelazquez@meei.harvard.edu (L.M.-V.); eric_pierce@meei.harvard.edu (E.A.P.); Jason_comander@meei.harvard.edu (J.I.C.); 2 Department of Ophthalmology and Vision Sciences, Temerty Faculty of Medicine, University of Toronto, Toronto, ON M5T 3A9, Canada

**Keywords:** sector retinitis pigmentosa, pericentral retinitis pigmentosa, rhodopsin, autosomal dominant, inherited retinal disease

## Abstract

Sector and pericentral are two rare, regional forms of retinitis pigmentosa (RP). While usually defined as stable or only very slowly progressing, the available literature to support this claim is limited. Additionally, few studies have analyzed the spectrum of disease within a particular genotype. We identified all cases (9 patients) with an autosomal dominant Rhodopsin variant previously associated with sector RP (*RHO* c.316G > A, p.Gly106Arg) at our institution. Clinical histories were reviewed, and testing included visual fields, multimodal imaging, and electroretinography. Patients demonstrated a broad phenotypic spectrum that spanned regional phenotypes from sector-like to pericentral RP, as well as generalized disease. We also present evidence of significant intrafamilial variability in regional phenotypes. Finally, we present the longest-reported follow-up for a patient with *RHO*-associated sector-like RP, showing progression from sectoral to pericentral disease over three decades. In the absence of comorbid macular disease, the long-term prognosis for central visual acuity is good. However, we found that significant progression of *RHO* p.Gly106Arg disease can occur over protracted periods, with impact on peripheral vision. Longitudinal widefield imaging and periodic ERG reassessment are likely to aid in monitoring disease progression.

## 1. Introduction

Retinitis pigmentosa (RP) is a group of syndromic and non-syndromic inherited retinal dystrophies characterized by progressive degeneration of the retina leading to nyctalopia and visual field defects. It is one of the leading causes of low-vision in adults, with a prevalence of approximately 1-in-2500 to 1-in-4000, and affecting more than two million people worldwide [1]. It is a genetically heterogenous disorder, with mutations in more than 75 genes known to cause non-syndromic RP alone (https://sph.uth.edu/retnet/ (accessed on 1 August 2021)). While in typical RP, retinal degeneration begins in the mid-periphery, leading to progressive visual field constriction and eventual loss of visual acuity [2], regional varieties such as pericentral RP [3,4] and sector RP [5] exist. Sector RP remains infrequently described because it is rare and patients may often not become symptomatic, given the limited regional nature of the visual field loss. When originally described in 1937, it was defined as a stationary variant [6]. Subsequent reports have suggested that sector RP is only very slowly progressive [7]. It has a tendency to affect the inferior and nasal quadrants with corresponding superior visual field defects [6,8,9]. Before the era of molecular genetic classification, it was appreciated that autosomal dominant retinitis pigmentosa (ADRP) could exist on a spectrum of regionality [8]; even then, intrafamilial variability was appreciated in the extent of anatomical regionality and penetrance of the RP phenotype, and had been noted by others [10,11].

Rhodopsin (*RHO*) was the first gene in which RP-causing mutations were identified [12,13]. In the early 1990s, it became appreciated that mutations in *RHO* were associated with ADRP, and that several distinct mutations showed regional anatomic and functional predilections [14,15,16,17,18]. Even the most common p.Pro23His mutation responsible for *RHO*-associated ADRP has shown regional phenotypes [9]. Recently, Georgiou and colleagues undertook a survey of the disease spectrum in molecularly-confirmed cases of sector-like RP [19], highlighting associated variants in a number of additional genes including *RPGR*, *USH1C*, *MYO7A, CDH23, EYS*, *IMPDH1*, *RP1*, and *RHO*. As discussed, while sector RP is generally thought of as a mild phenotype when compared to generalized RP, with a good prognosis for visual acuity in the long-term [20], there is a lack of natural history and longitudinal data on which to base our expectations of disease progression.

The *RHO* c.316G > A, p.Gly106Arg variant was first described as a cause of autosomal dominant sector RP in four patients with a distinct phenotype characterized by pigmentary changes in the inferior retina and corresponding visual field impairment in the superior hemisphere [18], with a good central visual prognosis. This inferior sectoral phenotype was subsequently seen in five other patients [19,21,22], including three from the same family [21]. A careful review of the reported cases, however, shows that at least one of these patients had more extensive near-peripheral anatomic involvement and evidence of a pericentral ring scotoma on visual fields [21]. Indeed, this *RHO* variant had also been reported in association with pericentral RP [3].

There is limited literature regarding the phenotypic spectrum of this *RHO* variant as well as limited data, including natural history data, on regional forms of RP more generally. We undertook a review of all patients at a single tertiary referral center with molecular confirmation of the *RHO* c.316G > A, p.Gly106Arg variant. We define a previously unrecognized spectrum of disease spanning the regional phenotypes of sector-like to pericentral RP but also including generalized RP. Fundus autofluorescence highlights these anatomical findings, and we present corresponding findings from psychophysical testing. We also present evidence of significant intrafamilial variability between three patients in a single family. Finally, we present the longest reported follow-up for a patient with *RHO*-associated sector-like RP in the literature, showing evidence of progression from sectoral to pericentral disease over three decades. In addition to expanding the phenotypic spectrum of disease for this *RHO* variant, our observations suggest caution when prognosticating and counselling patients about the risk of disease progression.

## 2. Materials and Methods

Our study adheres to the tenets of the Declaration of Helsinki and was approved by the Institutional Review Board of Massachusetts Eye and Ear. The genetic database of the Massachusetts Eye and Ear (MEE) Inherited Retinal Disorders Service was searched for individuals with the *RHO* c.316G > A, p.Gly106Arg variant. Genetic testing was performed either via a commercial CLIA-certified laboratory or in the genetic lab of the MEE Ocular Genomics Institute using previously described methods [23]. All individuals with this variant, and with at least one clinical evaluation at MEE, were included.

The clinical histories and evaluations of all identified patients were reviewed. Best-corrected visual acuity was measured using the Snellen chart, and visual fields were assessed using Goldmann kinetic perimetry. Full-field electroretinography (ERG) was conducted with a Burian-Allen contact lens on a custom ERG system [24]. In addition to fundus examination, multimodal imaging was performed including widefield fundus photography (Optos), widefield fundus autofluorescence (AF; Optos), and spectral-domain optical coherence tomography (SD-OCT; Spectralis, Heidelberg Engineering, Heidelberg, Germany). Depending on the year of assessment, not all imaging modalities were available for every patient at every visit.

## 3. Results

Nine individuals (six females; three males) with the *RHO* c.316G > A, p.Gly106Arg variant were identified. The findings of clinical assessments at presentation and last follow-up are summarized in Table 1 and organized by patient age at presentation. Patients were between 13 and 59 years of age of initial presentation. The age of onset was variable: whereas three patients (Cases 2, 5, 7) described symptoms beginning in childhood, two patients (Cases 8 and 9) did not experience symptoms until their 40 s. The youngest patient (Case 1) was asymptomatic at presentation, but all others were symptomatic. Common symptoms included delayed dark adaptation, nyctalopia, and awareness of peripheral visual field deficits. Four patients (Cases 3, 6, 7, 8) described an awareness of a pericentral visual field defect at presentation, while one patient (Case 4) described flashes in her superior visual field in both eyes that corresponded to a superior near-peripheral scotoma on visual field testing.

At the time of initial examination, best-corrected visual acuity was 20/20 in all eyes, with the exception of one eye with a comorbid macular hole (Case 5). Kinetic perimetry showed a range of findings from superior near or near-to-mid peripheral scotoma (3 patients), to pericentral ring scotoma (2 patients), to generalized mid-peripheral scotoma (2 patients). The youngest patient, who was asymptomatic, had normal perimetry.

**Figure 1:**
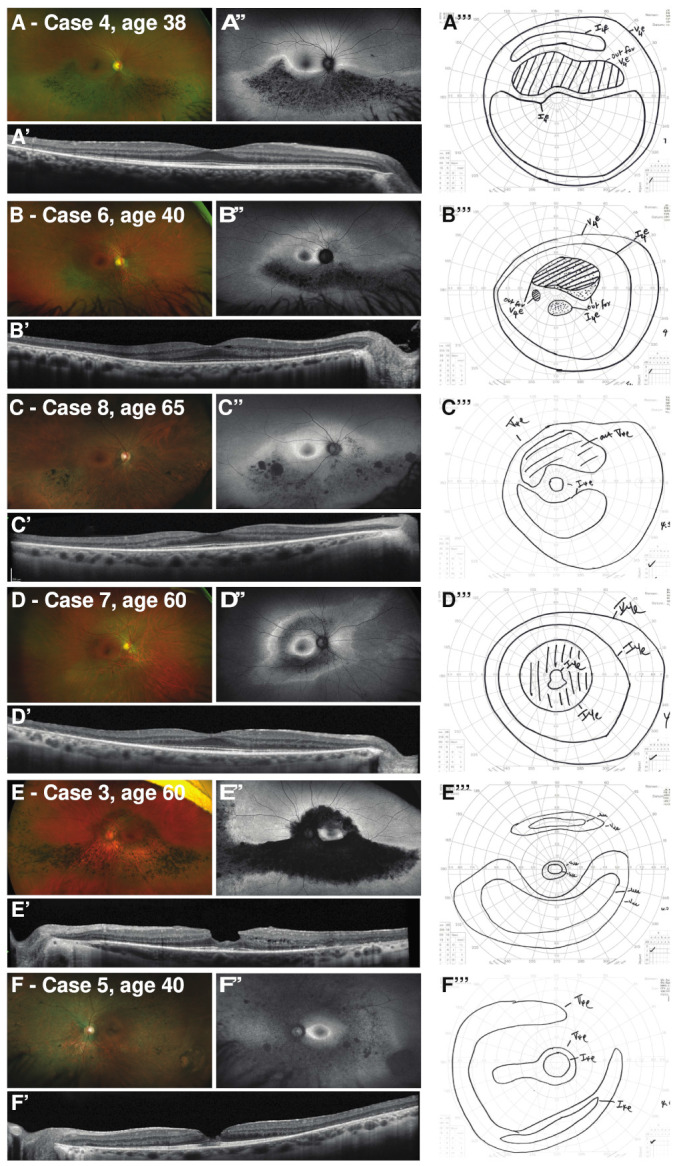
Spectrum of *RHO*-associated dystrophy caused by the p.Gly105Arg variant, ranging from sector-like RP to pericentral RP to generalized RP for 6 unrelated patients. (**A**–**F**) Widefield color images; (**A**’–**F**’) macular OCT centered on the fovea; (**A**’’–**F**’’) widefield fundus autofluorescence; and (**A**’’’–**F**’’’) manual Goldmann kinetic perimetry (V4e and I4e stimuli; scotoma shaded), for the corresponding eye. The case number and age of the patient is noted in the figure, and a description of corresponding clinical features and results of testing can be found in Table 1 and Table 2.

**Figure 2:**
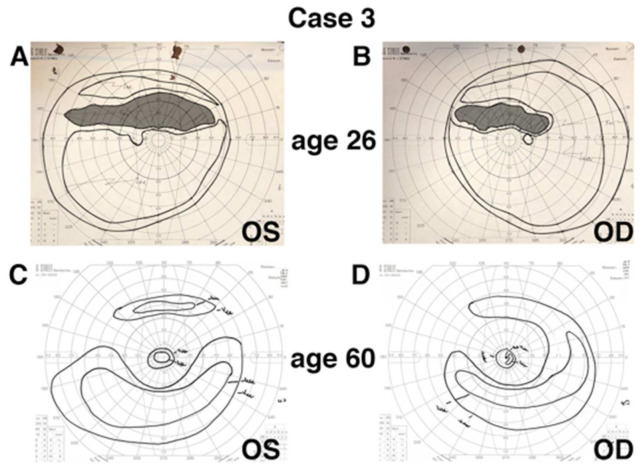
Perimetry reflecting progression of sector-like RP to pericentral RP in a patient after 34 years of follow-up. Manual Goldmann kinetic perimetry (V4e and I4e stimuli) are presented for the right and left eye at first presentation (age 26; isopter tracing highlighted) and at last follow-up (age 60) for Case 3. Perimetry at presentation (**A**,**B**) demonstrates a clear superior field scotoma, corresponding to the clinical description of sector RP at that evaluation. A pericentral pattern of field loss is present at last follow-up (**C**,**D**), corresponding to anatomic changes seen in the near periphery (see Figure 1); as well, a peripheral extension of the ring scotoma in the superior field corresponds to the sectoral band of atrophy visible across the inferior retina.

**Figure 3:**
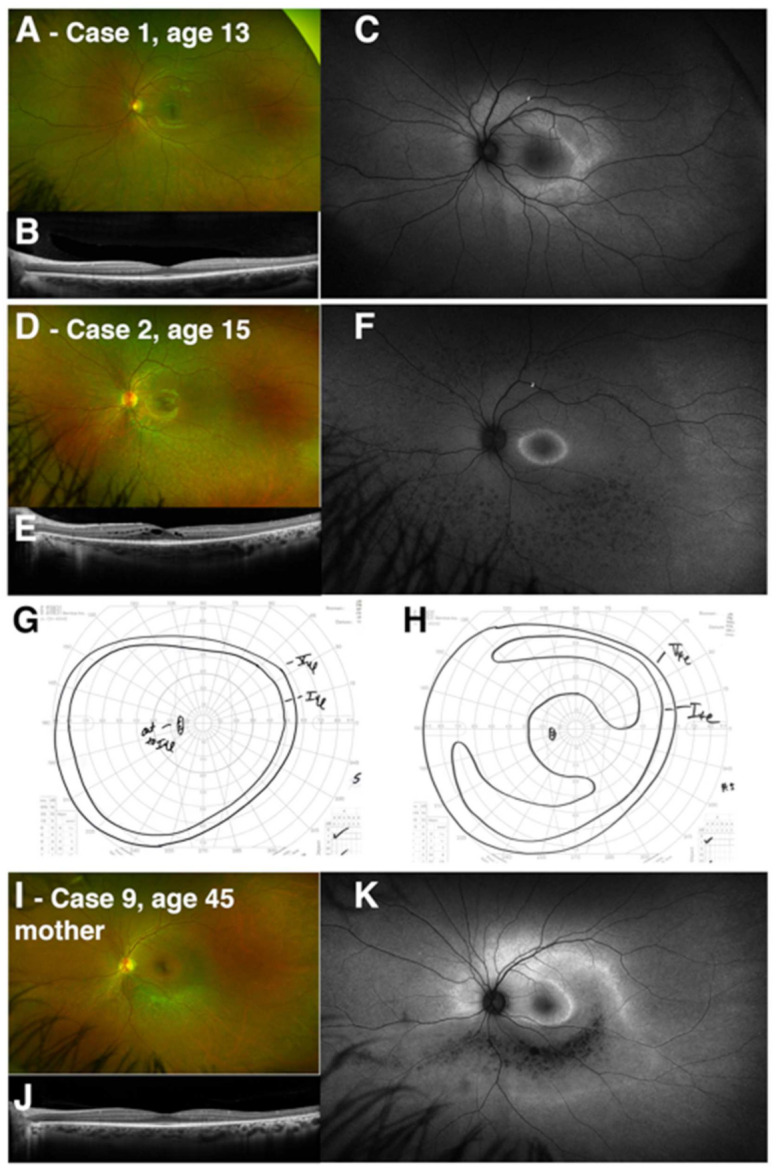
Intrafamilial variation in *RHO*-associated dystrophy caused by the p.Gly106Arg variant. Two siblings are presented with changes reflecting early and presymptomatic pericentral change (Case 1) and generalized RP (Case 2). (**A**,**D**) Widefield color images, (**B**,**E**) macular OCT line scan centered on the fovea, and (**C**,**F**) fundus autofluorescence are presented for the left eye for both cases. Manual Goldmann kinetic perimetry is presented from the left eye for both cases (**G**, Case 1; **H**, Case 2). The case number and age of the patient is noted in the figure, and a description of the corresponding clinical features and results of testing can be found in Table 1 and Table 2. Clinical imaging for the sibling’s mother (Case 9) is presented, including color imaging (**I**), OCT (**J**), and fundus autofluorescence (**K**), demonstrating a pericentral pattern of anatomic involvement, with a predominant inferior sectoral band of atrophy, as seen in other patients with p.Gly106Arg-associated dystrophy in this case series.

Multimodal imaging (fundus photography, OCT, fundus autofluorescence) showed anatomic features ranging from inferior sector-like atrophy and bone spicule pigmentation in an altitudinal pattern extending into the nasal periphery to near-to-mid-peripheral (i.e., pericentral) atrophy surrounding the macula with sparing of the far-periphery (Table 2; Figure 1). Seven cases from different families (Cases 2–8) showed anatomic involvement in spatial distributions correlating closely with perimetry findings (Figure 1, Figure 2 and Figure 3D–F,H). Three of these cases (Cases 3, 6, 8) showed combined anatomic features of both inferior sector-like and pericentral disease. In Case 3, the latter manifested as overt outer retinal atrophy on exam with accompanying findings on retinal imaging. In contrast, the pericentral involvement was more subtle on exam in Cases 6 and 8 and better-highlighted by abnormal macular autofluorescence and significant outer retinal atrophy on OCT. These features suggest a spectrum of disease connecting both phenotypes; the visual fields reflect this spectrum of pericentral ring scotoma combined with superior near-peripheral altitudinal scotoma. One case (Case 5) had anatomic findings characteristic of generalized RP.

Supplementary Figure S1 provides the ERG recordings for these cases at presentation and follow-up, where available; AF, autofluorescence; SD-OCT, spectral-domain optical coherence tomography; ffERG, full-field electroretinogram; IT, 30 Hz implicit time (normal: 25–32 ms); OD, right eye; OS, left eye; OU, both eyes; N/A, not available; CME, cystoid macular edema; * siblings; ** % of normal response indicated; ^†^ mother of Cases 1 and 2.

The electrophysiologic responses showed a similar spectrum of disruption (Table 2; Supplementary Figure S1). Patients with a sector-like or pericentral phenotype had ERGs characterized by normal to mildly-subnormal rod-isolated (scotopic dim-flash) and cone-isolated (30 Hz flicker) responses. Two patients, including the youngest patient who was asymptomatic, had normal ERG responses. In general, this is consistent with the spectrum of mild RP phenotypes as well as with previous reports [3,4]. The case with findings characteristic of generalized RP (Case 5) had the greatest reduction in rod- and cone-isolated responses. Delayed cone implicit time has been identified as a consistent feature of generalized RP [25], while in the presence of sector RP, cone implicit times have been reported to be normal [5]. This has been suggested to be a distinction between regional disease, versus generalized and progressive disease, with patients exhibiting normal cone implicit times suggested to have a better prognosis [26]. Implicit times for 30 Hz (cone-isolated) responses were measured (Table 2; Supplementary Figure S1). The delay in implicit time at last follow-up appears to increase with increasing anatomic burden of disease across cases (Figure 1), with the greatest delay in implicit time in typical generalized RP (Case 5).

Longitudinal assessment was available for three patients (Cases 3, 7, 8) with two showing stability. Case 7 was followed over 13 years, and showed no change in visual acuity, minimal-to-no change in a pericentral ring scotoma identified on perimetry, and minimal-to-no change in full-field ERG response amplitudes, with no change in cone implicit time. Case 8 similarly showed minimal progression over 6 years in perimetry and ERG response amplitudes; slight changes in visual acuity may have reflected the development of bilateral cataracts which were noted at the most recent follow-up.

In contrast, phenotypic evolution was seen in Case 3, who had 34 years of follow-up (Figure 2). She was diagnosed with sectoral RP at age 26 based on fundus findings, a visual field showing a superior field scotoma, and normal full-field ERG responses (Table 1 and Table 2). By age 60, her condition had progressed in both eyes to involve the circumferential near-periphery, giving a pericentral pattern of RP, with an associated decrease in her rod- and cone-isolated ERG responses. Notably, normal cone (30 Hz) implicit times at first presentation were not predictive of stationary disease in the long-term, as reflected by significantly delayed implicit times at last follow-up (Table 2; Supplementary Figure S1). While fundus autofluorescence and OCT were not available technologies at her first visit, her perimetry reflects those changes noted in her clinical evaluations (Figure 2A,B compared to Figure 2C,D). Changes in her best-corrected visual acuity were independent of these RP-associated findings, as she developed a macular hole in the right eye for which she underwent vitrectomy and membrane peeling (at age 42), and an epiretinal membrane with pseudohole in the left eye (untreated).

Finally, two cases (Cases 1 and 2) represent siblings who show significant intrafamilial variability (Figure 3A–H). The younger brother (Case 1, age 13) was asymptomatic and had only mild findings on fundus autofluorescence and OCT suggestive of early pericentral change. His older sister (Case 2, age 15) experienced nyctalopia and delayed dark adaptation, and had findings suggestive of more generalized, though as yet mild, RP. Retinal imaging was performed on their mother (Case 9, age 45). She was found to be a carrier of the *RHO* p.Gly106Arg variant only after her children’s diagnosis, leading to a limited evaluation focused on the pattern of retinal involvement. The mother described mild concerns with dark adaptation, and imaging demonstrated a pericentral pattern of autofluorescent change and hypoautofluorescence overlying the arcades, corresponding to outer retinal thinning and atrophy (Figure 3I–K). Full-field ERG and perimetry were not available.

## 4. Discussion

This investigation provides a dramatic presentation of the phenotypic spectrum of disease linking the diagnoses of two clinically-distinct presentations—sector and pericentral—of RP associated with the same *RHO* variant. By providing extended follow-up and widefield imaging, as well as psychophysical testing, we demonstrate the potential for significant variability in presentation and also progression in regional forms of RP. This highlights the limitations of using a rigid clinical classification schema to prognosticate disease progression in any individual patient. Furthermore, this variability in phenotype is not restricted to a spectrum of regional disease across unrelated patients or across time. The variability between genotype and phenotype is broader than we may have previously imagined. The cases presented of two siblings, close in age, from a parent with a pericentral distribution of retinal changes, show one child with regional pericentral changes, and the other with generalized and typical, though early, RP. The spectrum of disease severity is reflected not only in the anatomical predilection for atrophy, but also in the electrophysiologic recordings, which show a spectrum of photoreceptor impairment. This stands in contrast to previous, though older, reports of low intrafamilial variability in clinical classifications of autosomal dominant RP [8], and supports the more recent evidence of high intrafamilial variability seen in typical *RHO*-associated RP [27]. It is evident from reviewing the literature, however, that sector RP as described constitutes a heterogenous presentation. Some authors use it to refer to disease in which the ERG is normal and for which there is no progression into spared areas of the retina [5]. Others have used the term to refer to non-progressive disease, but which may include electrophysiologic dysfunction of unaffected regions [28,29]. But it has also been used to refer to disease that starts in one region of the retina, but is progressive and eventually affects the entire retina [14].

One limitation of our analysis was that our study only focused on cases of RP associated with the single *RHO* p.Gly106Arg point mutation. Indeed, only one other mutation involving rhodopsin codon 106, p.Gly106Trp has been identified in patients with RP [30]; no clinical information about the family with this mutation was provided. However, the regional predilection for sector pigmentary changes in the inferior retina, with predominantly superior visual field loss, has been observed in patients with *RHO* mutations affecting codons 17 [16], 58 [15], 182 [16], 190 [17], 267 [17] and in some patients with codon 23 mutations [14], including the common p.Pro23His [9] which can have a wide range of phenotypes [31]. Unfortunately, an analysis of the spectrum of disease across multiple families with these mutations and their phenotypic presentations, as presented in our report, has not been performed. Interestingly, one report suggested that unique point mutations in specific rhodopsin codons might lead to distinct RP phenotypes [17]: in one family with a p.Asp190Tyr mutation, the phenotype was one of severe and diffuse typical RP, while in another family with a p.Asp190Asn mutation, the phenotype was one typical of sector RP with an inferior regional predilection. Our report of a sibship with the same p.Gly106Arg point mutation and distinct regional versus generalized presentations argues against this as a general mechanism for phenotypic variability. An alternative hypothesis may be one of genetic modifiers that can affect the presentation and lead to interfamilial variability [27]. There are also several *RHO* variants that are rare in the general population but do not segregate with disease; while this suggests that these may be benign polymorphisms [32], it is possible that they could function as disease modifiers themselves.

Light exposure, as an environmental modifier, has been proposed given the inferior predilection for sector RP. It has been shown in animal models *RHO*-associated RP that light deprivation reduces the rate of retinal degeneration [33]. Consistently, even in those patients with pericentral RP seen in our study, the majority of the geographic burden of disease was found in the inferior hemisphere. As attractive as this hypothesis is, however, there may be intrinsic cell-specific differences in the superior and inferior human retina that may modify disease progression. Certainly this is true for other mammalian systems, including the mouse [34], where cone photoreceptors vary in their type and opsin expression between dorsal and ventral retina [35]. It is also true of models of retinal disease, such as mouse and rat. For example, the rate of cone inner segment loss in the albino Royal College of Surgeon’s (RCS) rate is greater in the ventral and temporal retina than dorsal retina [36,37]. Degenerative rosette formation forms preferentially in the ventral retinal of neural retinal leucine zipper (*NRL*)-/- mice, in which deletion of the rod-specific *NRL* gene results in specification of S-cone-like photoreceptors at the expense of rod photoreceptors [38]. Interestingly, light-induced oxidative stress is known to cause rapid loss of photoreceptors in the dorsal retina of albino rats, rather than the ventral retina [39].

All of the mutations listed above (including in codon 106) as described in the Human Gene Mutation Database (HGMD; www.hgmd.cf.ac.uk), based on their experimentally studied biochemical and cellular characteristics, as class 2 mutations [40]. That is, they are mutations that cause protein misfolding and instability, leading to protein retention in the endoplasmic reticulum (ER) [32]. Rhodopsin is an exquisitely sensitive molecule, and its structure involves a delicate balance of interactions which keep transmembrane domains intact and yet allow for complete receptor activation after absorption of a single photon by retinal. Rhodopsin folding and regulation by the ER are likely tightly regulated to allow only properly folded protein to reach the outer segments. Given the very high demands for expression and biosynthesis of rhodopsin in the human retina, this would lead to the production of a significant amount of defective protein, which may overwhelm the proteostatic mechanisms of the photoreceptor. These hypotheses as to the potential interplay of structural mutations with environmental modifiers are reviewed well elsewhere [32].

Finally, the importance of longitudinal follow-up for these patients cannot be understated. Our study includes three cases with long-term re-assessment, including one patient evaluated after more than 30 years of disease, which clearly demonstrates the evolution in her clinical and electrophysiologic findings. A recent study of the etiology of sector RP across a variety of genotypes was conducted by Georgiou et al. [19], including *RHO* variants, and suggested that any progression that was noted up to 6 years of follow-up was small in extent with little clinical impact. While we agree with the authors’ conclusion that the long-term prognosis for central visual acuity is good in many of these patients, significant progression in the disease with clinical and functional impact on peripheral vision can occur over longer periods in *RHO* p.Gly106Arg disease. The changes noted in our patients with protracted follow-up also support their hypothesis. The cross-sectional cases presented here suggest that widefield fundus autofluorescence in conjunction with perimetry offer valuable information about structure and function for longitudinal monitoring and counseling. Repeat ERG testing, while subject to more variability in responses, may be considered periodically as appropriate for individual patients.

## Figures and Tables

**Table 1:** Clinical Characteristics, Visual Acuity, and Visual Fields at Presentation and Last Follow-up.

Case Number	Research ID	Sex	Age of Symptom Onset	At Presentation				At Last Follow-Up			Additional Notable Diagnoses
				Age	Symptoms	Snellen BCVA	V4e Field Description	Age	Snellen BCVA	V4e Field Description	
1 *	OGI3683_0052104	M	N/A	13	None	20/20 OD 20/20 OS	Full OU	N/A	N/A	N/A	None
2 *	OGI3683_0052103	F	12	15	Nyctalopia, delayed dark adaptation	20/20 OD 20/20 OS	Full OU; I4e mid-peripheral relative scotoma OU	N/A	N/A	N/A	None
3	OGI992_001975	F	Early 20 s	26	Superior visual field defect	20/20 OD 20/20 OS	Superior near-peripheral scotoma OU	60	20/100 OD 20/20 OS	Pericentral ring scotoma	Macular hole OD (closed) Macular pseudohole OS
4	OGI3707_0052135	F	38	38	Flashes in superior visual field	20/20 OD 20/20 OS	Superior near-peripheral scotoma OU	N/A	N/A	N/A	None
5	OGI3706_0052134	M	Childhood	40	Delayed dark adaptation, blurry vision OD	20/100 OD 20/20 OS	Pericentral-to-mid-peripheral ring scotoma OU	N/A	N/A	N/A	Macular hole OD (open) Macular pseudohole OS
6	OGI3708_0052136	M	38	40	Blurry vision, near-peripheral field defect	20/20 OD20/20 OS	Superior near-peripheral scotoma OU, with inferior near-peripheral scotoma OS	N/A	N/A	N/A	X-linked deuteranomalous defect
7	OGI3705_0052133	F	Childhood	47	Nyctalopia, near-peripheral field defect	20/20 OD2 20/20 OS	Pericentral ring scotoma OU	60	20/20 OD 20/20 OS	Pericentral ring scotoma OU	None
8	OGI686_001369	F	47	59	Nyctalopia, near-peripheral field defect	20/20 OD 20/20 OS	Superior mid-peripheral scotoma OU	65	20/25 OD 20/40 OS	Superior mid-peripheral scotoma OU	None
9 ^†^	OGI3683_0052102	F	Mid-40 s	45	Mild delayed dark adaptation	20/20 OD 20/20 OS	N/A	N/A	N/A	N/A	None

BCVA, best-corrected visual acuity; M, male; F, female; OD, right eye; OS, left eye; OU, both eyes; N/A, not available; * siblings; ^†^ mother of Cases 1 and 2.

**Table 2:** Clinical Examination, Retinal Imaging, and ERG Testing at Presentation and Last Follow-up.

		At Presentation				At Last Follow-Up			
Case Number	Research ID	Fundoscopy	Fundus AF	SD-OCT	ffERG **	Fundoscopy	Fundus AF	SD-OCT	ffERG **
1 *	OGI3683_0052104	Normal macular and peripheral retinal pigmentation	Faint, diffuse hypoAF along arcades with surrounding hyperAF border in temporal perifovea forming partial ring	Preserved lamination in fovea and peripheral macula; subtle attenuation of ONL in temporal macula	Normal rod and cone responses. IT: 28 ms OD/28 ms OS	N/A	N/A	N/A	N/A
2 *	OGI3683_0052103	Mid-peripheral depigmentation and rare bone spicule pigmentation; mild vessel attenuation	Parafoveal hyperAF ring; mid-peripheral hypoAF; some far-anterior preserved AF	Preserved lamination in fovea; attenuation of ONL and loss of outer retinal banding in peripheral macula. Mild CME OD and mild-moderate CME OS.	Mildly subnormal, but readily detectable, rod-isolated (40% OD; 49% OS) and cone-isolated (42% OU) responses. IT: 38 ms OD/40 ms OS	N/A	N/A	N/A	N/A
3	OGI992_001975	Sectoral atrophy and bone spicule pigmentation inferior near-periphery and nasally	Unavailable	Unavailable	Normal rod and cone responses. IT: 32 ms OD/32 ms OS	Ring of near-peripheral atrophy overlying arcades with inferior and nasal extension into mid-periphery with bone spicule pigmentation. Foveal hypopigmentation OD.	Ring of pericentral hypoAF overlying arcades with inferior and nasal extension into mid- and far-periphery.Foveal hypoAF OD.	Foveal thinning and outer retinal atrophy with subretinal hyperreflective material OD. Macular pseudohole with epiretinal membrane, and preserved outer retinal banding in fovea, with small inner retinal pseudocysts in temporal parafovea OS. Attenuation of ONL and loss of outer retinal banding in peripheral macula OU.	Subnormal, but readily detectable, rod-isolated (46% OD; 52% OS) and cone-isolated (52% OD; 68% OS) responses. IT: 37 ms OD/34 ms OS
4	OGI3707_0052135	Sectoral atrophy and bone spicule pigmentation inferior near-periphery and nasally	Sectoral band of hypoAF in area of spicules in inferior near-periphery and nasally, with hyperAF border in inferior perifovea.	Preserved lamination in fovea; attenuation of ONL and loss of outer retinal banding in inferior macula.	Mildly subnormal, but readily detectable, rod-isolated (36% OD; 39% OS) and cone-isolated (78% OD; 66% OS) responses. IT: 30 ms OD/30 ms OS	N/A	N/A	N/A	N/A
5	OGI3706_0052134	Near- to mid-peripheral atrophy with sparse bone spicule pigmentation and far-peripheral sparing. Macular hole OD; macular pseudohole OS.	Near- to mid-peripheral hypoAF with far-peripheral preserved AF. Perifoveal hyperAF ring OU.	Full-thickness macular hole OD; macular pseudohole OS. Attenuation of ONL and loss of outer retinal banding in peripheral macula.	Subnormal, but detectable, rod-isolated (20% OD; 17% OS) and cone-isolated (58% OD; 52% OS) responses. IT: 37 ms OD/38 ms OS	N/A	N/A	N/A	N/A
6	OGI3708_0052136	Sectoral atrophy and sparse bone spicule pigmentation inferior near-periphery and nasally	Sectoral band of hypoAF in inferior near-periphery and nasally. Ring of AF change overlying arcades with hyperAF ring in perifovea.	Preserved lamination in fovea; attenuation of ONL and loss of outer retinal banding in peripheral macula.	Normal rod and cone responses. IT: 35 ms OD/34 ms OS	N/A	N/A	N/A	N/A
7	OGI3705_0052133	Near- to mid-peripheral atrophy with rare bone spicule pigmentation and far-peripheral sparing.	Unavailable	Unavailable	Subnormal, but readily detectable, rod-isolated (63% OD; 48% OS) responses. Normal cone-isolated responses. IT: 33 ms OD/33 ms OS	Near- to mid-peripheral atrophy with rare bone spicule pigmentation and far-peripheral sparing.	Near- to mid-peripheral hypoAF with far-peripheral preserved AF. Perifoveal hyperAF ring OU.	Preserved lamination in foveal; attenuation of ONL and loss of outer retinal banding in peripheral macula.	Subnormal, but readily detectable, rod-isolated (52% OD; 46% OS) responses. Normal cone-isolated responses. Rod-isolated responses OD slightly diminished compared to presentation.IT: 31 ms OD/33 ms OS
8	OGI686_001369	Sectoral atrophy and bone spicule pigmentation inferior near-periphery and nasally	Unavailable	Preserved lamination in fovea; attenuation of ONL and loss of outer retinal banding in peripheral macula.	Subnormal, but readily detectable, rod-isolated (55%) responses OD and normal responses OS. Subnormal but readily detectable cone-isolated (64% OU) responses. IT: 37 ms OD/35 ms OS	Sectoral atrophy and bone spicule pigmentation inferior near-periphery and nasally	Sectoral band of hypoAF in inferior near-periphery and nasally. Ring of AF change overlying arcades with hyperAF ring in perifovea.	Preserved lamination in fovea; attenuation of ONL and loss of outer retinal banding in peripheral macula.	Subnormal, but readily detectable, rod-isolated (36% OD; 34% OS) and cone-isolated (62%; 74%) responses. IT: 34 ms OD/35 ms OS
9 ^†^	OGI3683_0052102	Sectoral atrophy and sparse bone spicule pigmentation inferior near-periphery and nasally	Sectoral band of hypoAF in inferior near-periphery and nasally. Ring of AF change overlying arcades with hyperAF ring in perifovea.	Preserved lamination in fovea; attenuation of ONL and loss of outer retinal banding in peripheral macula.	N/A	N/A	N/A	N/A	N/A

Supplementary Figure S1 provides the ERG recordings for these cases at presentation and follow-up, where available; AF, autofluorescence; SD-OCT, spectral-domain optical coherence tomography; ffERG, full-field electroretinogram; IT, 30 Hz implicit time (normal: 25–32 ms); OD, right eye; OS, left eye; OU, both eyes; N/A, not available; CME, cystoid macular edema; * siblings; ** % of normal response indicated; † mother of Cases 1 and 2

## Data Availability

Informed consent was obtained from all subjects involved in the study.

## Supplementary Materials

The following are available online at https://www.mdpi.com/article/10.3390/genes12121853/s1, Figure S1: Full-field electroretinography (ERG) responses for patients with *RHO*-associated dystrophy caused by the p.Gly106Arg variant.
