# Supplementary material for: Beyond Sector Retinitis Pigmentosa: Expanding the Phenotype and Natural History of the Rhodopsin Gene Codon 106 Mutation (Gly-to-Arg) in Autosomal Dominant Retinitis Pigmentosa
Source: Genes (Basel). 2021 Nov 23;12(12):1853. doi: 10.3390/genes12121853 (PMC8701931; doi:10.3390/genes12121853)
Supplement: Supplementary file 1 [file genes-12-01853-s001.zip › Supplementary Figure S1.pdf]

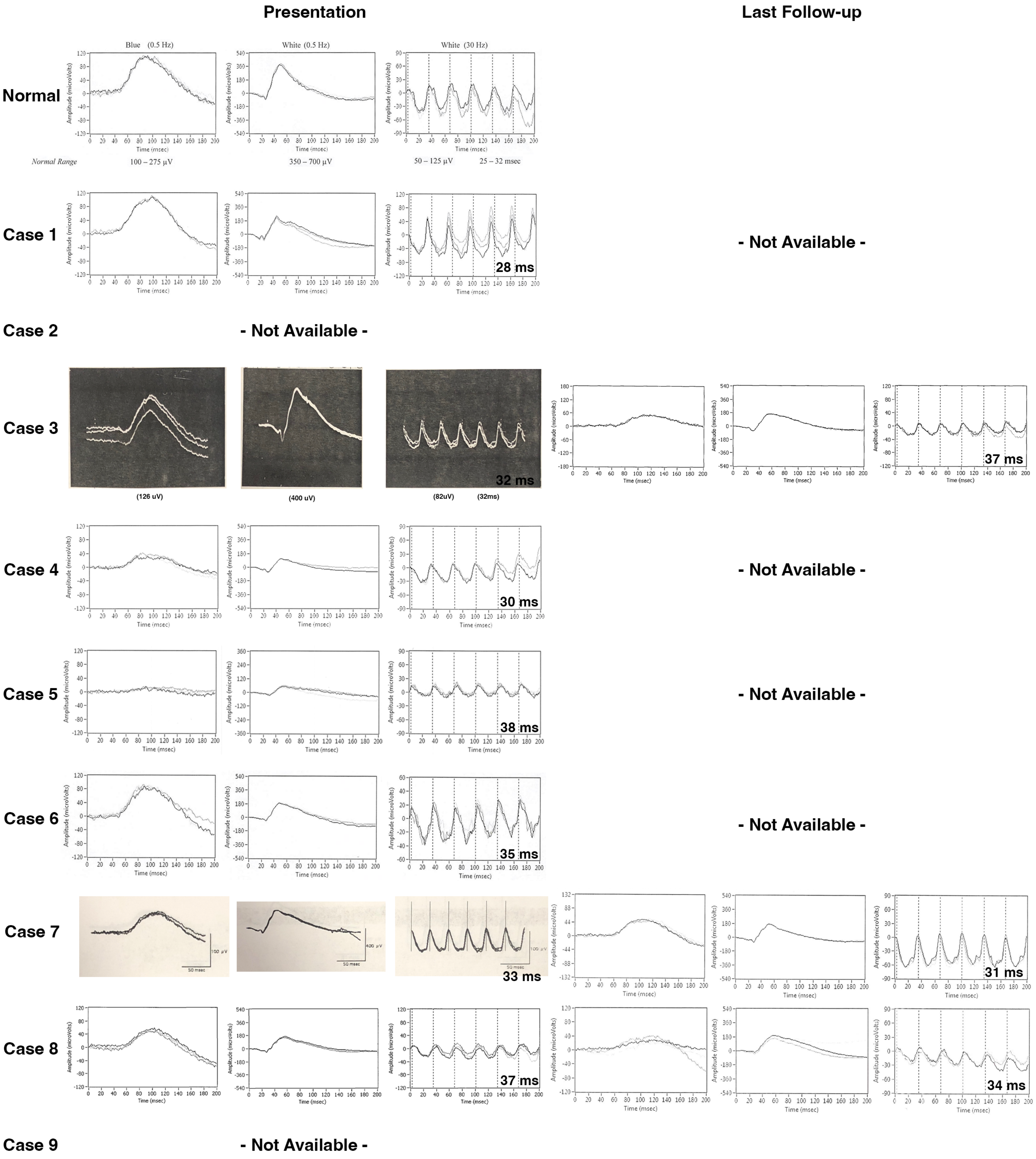
Presentation
Last Follow-up
Blue (0.5 Hz)
White (0.5 Hz)
White (30 Hz)
Normal
Normal Range
100 – 275 μV
350 – 700 μV
50 – 125 μV
25 – 32 msec
Case 1
28 ms
- Not Available -
Case 2
- Not Available -
Case 3
32 ms
(126 uV)
(400 uV)
(82uV)
(32ms)
37 ms
Case 4
30 ms
- Not Available -
Case 5
38 ms
- Not Available -
Case 6
35 ms
- Not Available -
Case 7
33 ms
31 ms
Case 8
37 ms
34 ms
Case 9
- Not Available -
Amplitude (microVolts)
Time (msec)
