# Supplementary material for: Beyond Sector Retinitis Pigmentosa: Expanding the Phenotype and Natural History of the Rhodopsin Gene Codon 106 Mutation (Gly-to-Arg) in Autosomal Dominant Retinitis Pigmentosa
Source: Genes (Basel). 2021 Nov 23;12(12):1853. doi: 10.3390/genes12121853 (PMC8701931; doi:10.3390/genes12121853)
Supplement: Supplementary file 1 [file genes-12-01853-s001.zip › Supplementary Figure S1 caption.pdf]

genes

MDPI

SUPPLEMENTARY FIGURE CAPTIONS

Supplementary Figure S1. Full-field electroretinography (ERG) responses for patients with *RHO*-associated dystrophy caused by the p.Gly106Arg variant. The traces for rod-isolated (dark-adapted, dim-blue 0.5 Hz), combined rod and cone (dark-adapted, white 0.5 Hz), and cone-isolated (white 30 Hz flicker) responses are provided, along with representative normal responses. For patients who had longitudinal follow-up, traces at last follow-up are provided, as outlined in Table 2. Raw traces for Case 2 are not available, though a summary of the reported responses from an outside ERG are included in Table 2. An ERG was not performed in Case 9 as described in the text. The implicit times for the 30 Hz flicker recordings are provided on the traces.
